# A mammalianized synthetic nitroreductase gene for high-level expression

**DOI:** 10.1186/1471-2407-9-301

**Published:** 2009-08-27

**Authors:** Maik Grohmann, Nils Paulmann, Sebastian Fleischhauer, Jakob Vowinckel, Josef Priller, Diego J Walther

**Affiliations:** 1 Department of Human Molecular Genetics, Max Planck Institute for Molecular Genetics, Ihnestrasse 73, 14195 Berlin, Germany; 2 Department of Biology, Chemistry, Pharmacology, Free University Berlin, Takustrasse 3, 14195 Berlin, Germany; 3 Neuropsychiatry and Laboratory of Molecular Psychiatry, Charité-Universitätsmedizin Berlin, Charitéplatz 1, 10117 Berlin, Germany; 4 Department of Life Sciences and Technology, Beuth University of Applied Sciences, Luxemburger Strasse 10, 13353 Berlin, Germany

## Abstract

**Background:**

The nitroreductase/5-(azaridin-1-yl)-2,4-dinitrobenzamide (NTR/CB1954) enzyme/prodrug system is considered as a promising candidate for anti-cancer strategies by gene-directed enzyme prodrug therapy (GDEPT) and has recently entered clinical trials. It requires the genetic modification of tumor cells to express the *E. coli *enzyme nitroreductase that bioactivates the prodrug CB1954 to a powerful cytotoxin. This metabolite causes apoptotic cell death by DNA interstrand crosslinking. Enhancing the enzymatic NTR activity for CB1954 should improve the therapeutical potential of this enzyme-prodrug combination in cancer gene therapy.

**Methods:**

We performed *de novo *synthesis of the bacterial nitroreductase gene adapting codon usage to mammalian preferences. The synthetic gene was investigated for its expression efficacy and ability to sensitize mammalian cells to CB1954 using western blotting analysis and cytotoxicity assays.

**Results:**

In our study, we detected cytoplasmic protein aggregates by expressing GFP-tagged NTR in COS-7 cells, suggesting an impaired translation by divergent codon usage between prokaryotes and eukaryotes. Therefore, we generated a synthetic variant of the nitroreductase gene, called *ntro*, adapted for high-level expression in mammalian cells. A total of 144 silent base substitutions were made within the bacterial *ntr *gene to change its codon usage to mammalian preferences. The codon-optimized *ntro *either tagged to *gfp *or *c-myc *showed higher expression levels in mammalian cell lines. Furthermore, the *ntro *rendered several cell lines ten times more sensitive to the prodrug CB1954 and also resulted in an improved bystander effect.

**Conclusion:**

Our results show that codon optimization overcomes expression limitations of the bacterial *ntr *gene in mammalian cells, thereby improving the NTR/CB1954 system at translational level for cancer gene therapy in humans.

## Background

Cancer is the second most frequent cause of death in developed countries and a leading cause of death in the world. Fifty-eight million people died worldwide in 2005 and cancer accounted for 7.6 million (13%) of these deaths [1]. Twelve million people were newly diagnosed with cancer last year and the number is prognosed to continue rising with estimated up to 26 million new cases in 2030 [2]. Thus, novel and effective anti-cancer therapies are needed to encounter this extending disease.

Conventional cancer treatment involves chemotherapy, which is able to cure a certain number of cancers, such as leukemia. However, the chemotherapeutic effect on solid tumors is often only transient [3]. Chemotherapeutic drugs interfere with a broad spectrum of intracellular processes, but most of them target proliferating cells in general by inhibition of DNA synthesis or DNA damage. Thus, cancer drugs lack tumor specificity and nearly all organs become affected after systemic application making chemotherapy highly dose-limited. However, high drug concentrations are required to antagonize tumor development efficiently and often several chemotherapeutics need to be combined to circumvent possible drug resistance of cancer cells [3].

Gene-directed enzyme prodrug therapy (GDEPT) is a promising alternative approach for cancer treatment [4]. The principle of GDEPT involves the genetic modification of tumor cells to produce enzymes capable of metabolizing nontoxic prodrugs into potent cytotoxins. This offers the potential for much higher intratumoral cytotoxin concentrations than obtained after systemic application of the active cytotoxic species itself. Moreover, the specific activation of chemotherapeutic prodrugs in tumor cells *in situ *circumvents the dose-limiting systemic cytotoxicity on normal tissues, as observed in conventional chemotherapy. For this purpose, a number of different enzyme/prodrug combinations have been established recently [4,5].

The most extensively studied prodrug-activating enzyme is the *Herpes simplex virus *type 1 thymidine kinase, which activates the antiviral agent ganciclovir by phosphorylation [6]. Another enzyme, the *Escherichia coli *cytosine deaminase, catalyzes the deamination of 5-fluorocytosine to 5-fluorouracil, which is the favored chemotherapeutic drug in many gastrointestinal malignancies [4,7]. The active species of both enzyme/prodrug systems are nucleotide analogues that interfere with DNA synthesis causing chain termination and cell death exclusively in proliferating cells. These antimetabolite-producing systems have been successfully used in growing cell cultures and cancer tumor models [4,7]. However, their clinical applicability in gene therapy has been challenged in phase I and II trials by the fact that in human tumors non-dividing cells are much more abundant than proliferating cells, rendering most of the tumor cells insensitive to the prodrug [8]. Thus, alternative enzyme/prodrug combinations, which do not discriminate between dividing and non-dividing cells, for example by generating alkylating agents, are more suitable for gene therapy approaches.

In 1969 Cobb *et al*. reported that rat Walker 256 carcinoma cells are exceptionally sensitive to the weak monofunctional alkylating agent 5-(azaridin-1-yl)-2,4-dinitrobenzamide (CB1954) in comparison with Chinese hamster V79 cells [9]. This cytotoxicity was subsequently found to depend on the enzyme DT diaphorase in Walker tumor cells, which catalyzes the aerobic reduction of the 4-nitro group of CB1954 in the presence of NADH or NADPH [10]. The resulting 4-hydroxylamine derivative undergoes a further non-enzymatic reduction with cellular thioesters to become a potent DNA crosslinking agent [10]. As this DNA damage is poorly repaired, CB1954 treatment results in cell death via apoptosis in a p53-independent manner [4,11,12]. However, human cell lines are less sensitive to CB1954 due to a far lower activity of human DT diaphorase compared with the rat enzyme [10]. This fact, together with the identification of a bacterial nitroreductase (NTR) in *E. coli*, which bioactivates CB1954 about 60 times more efficiently than the Walker DT diaphorase, then increased the potential for application of CB1954 as an anti-tumor agent in humans [10].

Consistently, the specific cell ablation in different tissues, for example in mammary gland and the brain, has proven the potent applicability of the NTR/CB1954 system in transgenic animal models [11,13-15]. Nevertheless, high prodrug dosages are required to obtain the desired cell ablation resulting in side effects, such as considerable weight loss and testis degeneration (own observations).

Recent investigations have focused on the optimization of the NTR/CB1954 system by site-directed mutagenesis of the enzymes catalytic core [16,17]. However, the prodrug-metabolizing activity depends also on the amount of functional protein in the cell. The expression of prokaryotic genes in eukaryotes is often hampered by differential codon usage, whose pattern is not conserved in taxonomically distant species and specific biases exist [18,19]. The bacterial codon usage was shown to affect recombinant protein translation in mammals [20,21]. Therefore, codon optimization has been applied to improve the expression for a variety of bacterial genes in eukaryotes [20,21].

Here we show that expression of a *gfp*-tagged *E. coli ntr *gene (*gntr*) in mammalian cells is accompanied by aggregation of the recombinant fusion protein. A synthetic version, termed *gntro*, with silent mutations that adapt codon usage to mouse preferences showed higher protein levels and largely enhanced sensitivity to the prodrug CB1954 after expression in different mammalian cell lines.

## Methods

### Plasmids (constructs)

**Figure 1:**
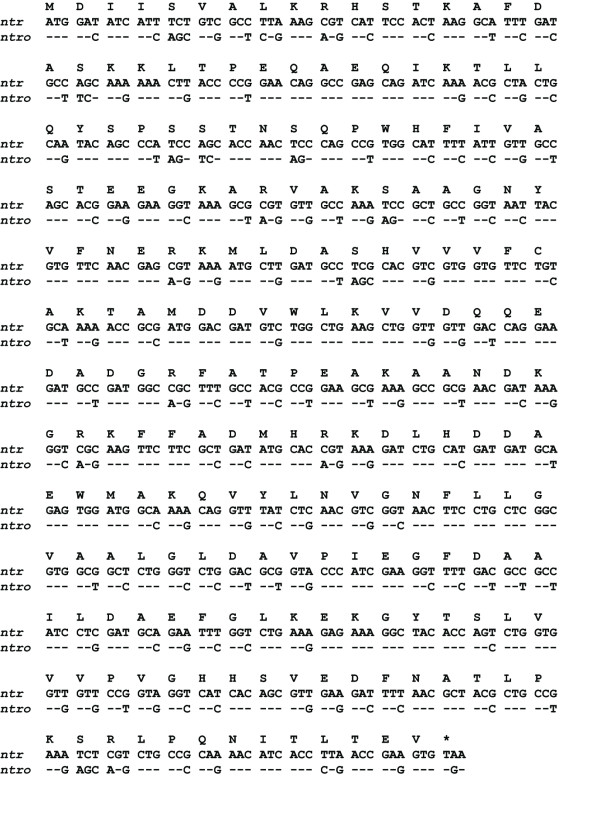
**Nucleotide sequence of nitroreductase cDNA and the deduced amino acid sequence**. The point mutations of *ntro *are shown below the substituted nucleotides of *ntr*. The corresponding amino acid is indicated above each codon.

The nitroreductase gene (*nfsB, ntr*) was amplified from *E. coli *DH5α genomic DNA by PCR with the primers 5'-GTCTTTATGGATATCATTTCTGTCG-3' (forward) and 5'-AGAGAGAATTACACTTCGGTTAAGG-3' (reverse). The obtained 654 bp amplicon was subcloned into pGEM^®^-T easy (Promega) and confirmed by sequencing. The codon-optimized *ntro *gene (Figure 1) was custom-synthesized by GeneScript (NJ, USA) and provided into the pUC57 subcloning vector. Codon usage tables were obtained from http://www.kazusa.or.jp[22]. To express nitroreductase with a N-terminal GFP-tag both genes were cut out with *Eco*R I from the respective source vectors and cloned into pEGFP-C1 (Clontech) using *Eco*R I restriction sites. The obtained constructs pGNTR and pGNTRo slightly differed in linker sequence (5 of 17 amino acids) and were used for all cell culture experiments. Amino- and carboxyterminally *c-myc*-tagged *ntr *constructs were generated by PCR-amplification from pGEM-NTR and pUC57-NTRo using either the primer pairs 5'-GGATCCATGGATATCATTTCTGTCG-3'/5'-AAGCTTTTACACTTCGGTTAAGGTG-3' (pMycNTR) and 5'-GGATCCATGGACATCATCAGCGTGG-3'/5'-AAGCTTTCACACC TCGGTCAGGG-3' (pMycNTRo) or 5'-GGATCCGCCGCCATGGATATCATTTCTG-3'/5'-AAGCTTCACTTCGGTTAAGGTGATGTTTTG-3' (pNTR-myc) and 5'-GGATCCGCCGC CATGGACATCATCAGCG-3'/5'-AAGCTTCACCTCGGTCAGGGTGATGTTC-3' (pNTRo-myc), respectively. The primers provided 5'-*Bam*H I and 3'-*Hin*d III restriction sites for subsequent cloning. All amplicons were subcloned into pGEM^®^-T easy and confirmed by sequencing. To express N-terminally-tagged NTR, the amplicons were cloned into pCMV-Tag3B (Stratagene) using *Bam*H I and *Hin*d III restriction sites. For the expression of the C-terminally-tagged NTR, the corresponding amplicons were cloned into pcDNA3.1/myc-His (-) A (Invitrogen), using the same strategy.

### Cell lines and culture conditions

Green monkey kidney (COS-7), human embryonic kidney (HEK-293), human neuroblastoma (SH-SY5Y), human small cell lung carcinoma (SHP-77), human osteosarcoma (U-2 OS), mouse fibroblast (3T3-L1), and mouse mastocytoma (P815) cells were maintained under standard conditions at 37°C and 5% CO_2 _in DMEM or RPMI (SHP-77 and P815) supplemented with 10% fetal bovine serum and antibiotics. COS-7 cells were transfected with the DEAE-dextran pretreatment method. Transiently transfected COS-7 cells were either used for cytotoxicity assays 24 hours after transfection by cultivating them with different CB1954 concentrations or for Western blot analysis at indicated time points. To establish stable cell lines, cells were transfected with DreamFect (OZ Biosciences) according to the manufacturer's instructions and selected in presence of 500 μg/ml G418 for 2 weeks. After selection, G418-resistent cells were pooled and FACS-sorted to purity using GFP as a fluorescence marker.

### MTT assay

Stable cells were seeded in quadruplicate on 24-well plates (2*10^5 ^cells/well) in DMEM without Phenol Red supplemented with different CB1954 concentrations. MTT solution (3-(4,5-dimethylthiazol-2-yl)-2,5-diphenyltetrazolium bromide) was added to each well after 48 hours to a final concentration of 0.5 mg/ml and cells were further incubated for 2 hours. Cells were detached, washed with PBS and suspended in 1 ml MTT stop solution (0.04 M HCl in isopropanol) to solubilize formazan crystals. After centrifugation supernatants were transferred in duplicate to a 96-well plate and extinctions were measured at 570 nm.

### Cell counting

Transiently transfected COS-7 cells were seeded in triplicate on a 96-well plate with the indicated concentrations of CB1954. The surviving green cells were counted after 48 hours of treatment within a field of view at 10× magnification using an Axiovert 40 microscope (Zeiss). A total of six randomly chosen fields were counted per experimental condition.

### Immunoblot analysis

After washing with PBS, cells were incubated on ice with RIPA lysis buffer (50 mM Tris-HCl pH 8.0; 150 mM NaCl; 1% NP-40; 0.5% DOC; 0.1% SDS) supplemented with protease inhibitors (Roche) for 10 min. Soluble fractions of cellular lysates were obtained by centrifugation at 20000 × *g *and 4°C for 5 min. Protein concentrations were determined using Bradford reagent (Sigma). Cellular lysates were separated by 12% SDS-PAGE and transferred to nitrocellulose (Amersham). Membranes were blocked with 3% BSA in phosphate-buffered saline (PBS) containing 0.1% Tween-20 and then probed sequentially with monoclonal mouse anti-GFP (1:2000, Cat. No. 1814460, Roche) or c-myc (1:500, sc-40, Santa Cruz Biotechnology) antibodies and the corresponding HRP-conjugated goat anti-mouse antibody (1:60000, A0168, Sigma). Loading of equal amounts of protein was confirmed by reprobing with polyclonal goat anti-actin (1:5000, sc-1616, Santa Cruz Biotechnology) and donkey anti-goat-HRP antibodies (1:60000, sc-2020, Santa Cruz Biotechnology). Immunoreactive bands were visualized with ECL PLUS (Perkin-Elmer).

### Fluorescence microscopy

Transiently transfected COS-7 cells were seeded on glass cover slips and cultivated in presence of various CB1954 concentrations for 24 hours. Cells were then fixed with 3.7% formaldehyde in PBS for 10 min. Genomic DNA was stained with DAPI (1 μg/ml in PBS), washed with PBS and mounted in Mowiol. Specimens were analyzed with 10× and 63× planapochromat objectives on an Axiovert 200 M microscope (Zeiss) equipped with epifluorescence optics.

### Statistical analysis

All data are presented as means ± SEM and two-tailed unpaired Student's t-test was used to compare data sets. Values of p < 0.05 were considered as statistically significant.

## Results

### Expression of *gntr *in COS-7 cells results in protein aggregation

**Figure 2:**
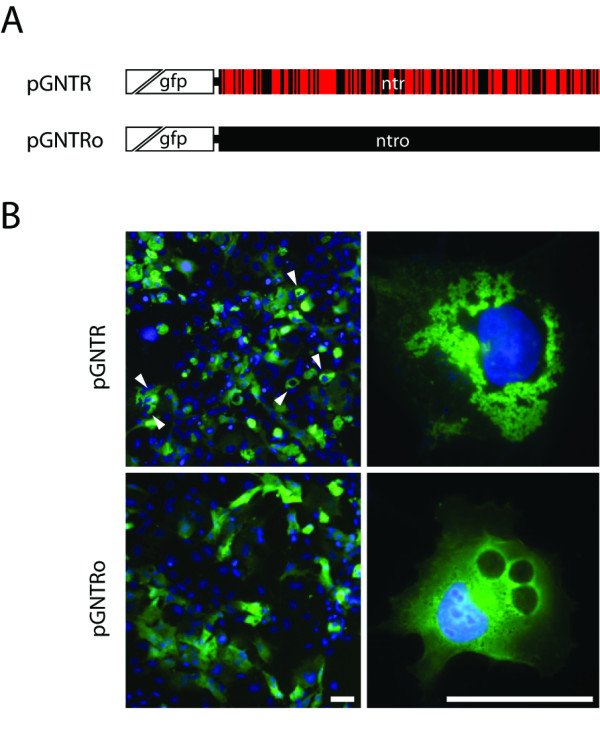
**The expression of the bacterial nitroreductase gene in COS-7 cells results in protein aggregation**. (A) Schematic representation of the coding regions concerning GNTR expression in pGNTR and pGNTRo constructs, respectively. The wild type *ntr *gene and the codon-optimized *ntro *gene were N-terminally fused to *gfp *providing a marker for NTR expression. The bacterial *ntr *sequence contains several critical codons (red bars) for the expression in mammalian cells. In pGNTRo all critical *ntr *codons have been adapted to the preferred codon usage in mouse to ensure optimal expression in mammalian cells. (B) Perinuclear structures (white arrowheads) are present in approximately 10% of pGNTR-transfected COS-7 cells, indicating protein aggregation 48 hours after transfection. Protein aggregation was not present in *gntro*-expressing cells after codon optimization. GFP-labeled nitroreductase (green) and DAPI-stained DNA (blue) are shown in overlay. Scale bar = 50 μm.

To test the efficacy of bacterial *ntr *gene expression in mammalian cell lines, we generated a *gfp/ntr *fusion construct (pGNTR) that facilitates the detection of NTR protein expression either by fluorescence microscopy or immunodetection (Figure 2A). Interestingly, we could detect perinuclear protein aggregates in the cytoplasm of approximately 10% of the pGNTR-transfected COS-7 cells 48 hours after transfection (Figure 2B). Thus, the question arose whether the divergent codon usage of prokaryotes and eukaryotes was responsible for the aggregates as approximately 60% of the bacterial *ntr *codons are underrepresented in mammalian genomes (e.g. *Mus musculus*; Figure 2A and Additional file 1) [22].

**Figure 3:**
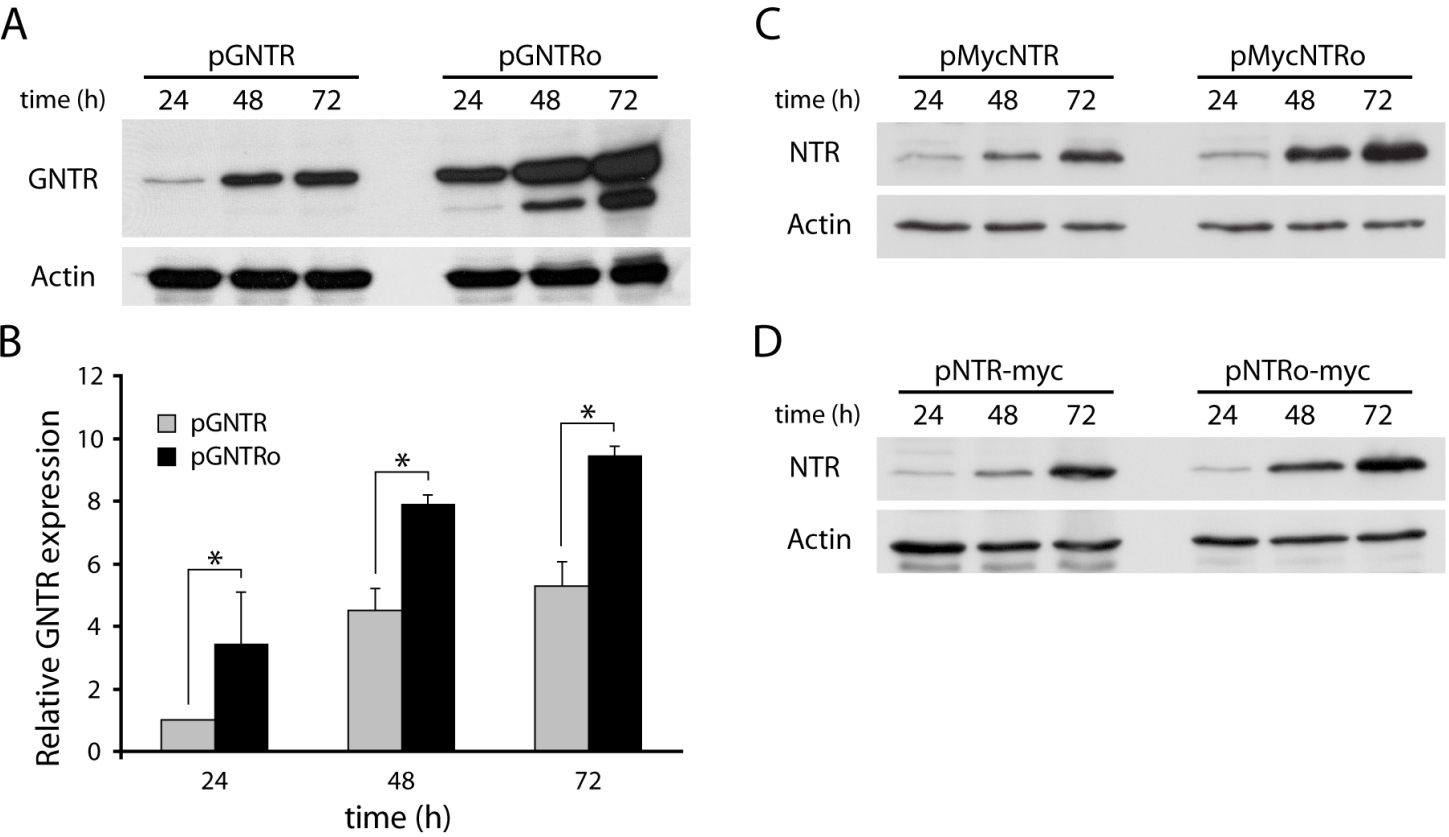
**The nitroreductase expression in COS-7 cells is improved by codon usage optimization**. (A) At the indicated time points cleared cellular lysates (20 μg) of pGNTR and pGNTRo-transfected cells were analyzed by immunoblotting using anti-GFP and anti-actin (loading control) antibodies. (B) Densitometric quantification of GNTR protein levels normalized to actin. Combined data of two independent experiments are represented as means ± SEM. *: p < 0.05; n = 2. (C, D) Consistently, the amino- (C) and carboxyterminally *c-myc*-tagged (D) *ntro *showed similarly improved expression levels as compared with the corresponding *ntr *constructs.

To test our hypothesis, we designed a synthetic version of the *ntr *gene, in which the rare codons for mammalian expression of the wild type *E. coli *gene were replaced by codons preferentially used in mammalian genomes (Figure 1). A total of 144 base substitutions in 124 codons were introduced without changing its amino acid sequence. The resulting codon-optimized *ntr *version (*ntro*) was fused to *gfp *and the corresponding pGNTRo construct (Figure 2A) was used to transfect COS-7 cells to directly compare *gntro *expression with *gntr*. Indeed the cytoplasmic aggregates evident for *gntr*-expressing cells could no longer be detected in cells expressing *gntro *(Figure 2B). Furthermore, the GNTR expression was remarkably improved after codon usage optimization to ~3-fold higher protein levels 24 hours after transfection (Figures 3A and 3B). The elevated *gntro *expression was stable throughout 48 to 72 hours with an average expression level 2-fold higher than of the wild type gene. In order to exclude any artefactual expression difference caused by the aminoterminal GFP-tag, amino- and carboxyterminal *c-myc *constructs were generated and tested by immunoblotting (Figures 3C and 3D). All codon-optimized expression constructs proved to lead to higher NTR levels as compared with the wild type cDNAs, using several independent batches of plasmid DNA.

### Codon optimization increases the sensitivity to CB1954

**Figure 4:**
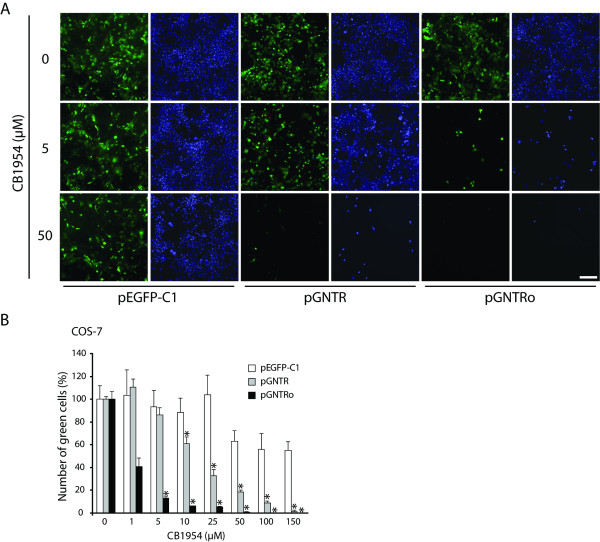
**Codon optimization increases the cytotoxicity of the NTR/CB1954 system in transiently transfected cells**. (A) COS-7 cells, transfected with pGNTR, pGNTRo or pEGFP-C1 (control) were cultivated in presence of increasing CB1954 concentrations. The survival of transfected cells (green) was analyzed by fluorescence microscopy 24 hours after CB1954 treatment. The DNA was stained with DAPI (blue). Scale bar = 100 μm. (B) The prodrug-mediated cell death was quantified by counting GNTR-expressing cells (green) 48 hours after treatment with CB1954. Cells expressing *gntro *were nearly fully depleted at concentrations below 25 μM, whereas a similar effect in *gntr*-expressing cells was observed only at CB1954 concentrations higher than 100 μM. Shown is one representative experiment out of two repetitions. *: p < 0.05; n = 3.

To test the enzymatic activity of GFP-tagged nitroreductase, we performed cytotoxicity assays to determine the sensitivity of both GNTR-expressing cell lines to the prodrug CB1954 (Figure 4A). Transfected COS-7 cells were treated with different concentrations of CB1954 for 24 hours. Fluorescence microscopy revealed efficient ablation of *gntr*-expressing cells by CB1954 at 50 μM. However, similar results were observed already at the 10 times lower prodrug concentration of 5 μM in cells expressing the optimized *gntro *gene. Control cells expressing *gfp *were not affected by 5 μM CB1954, but interestingly an unspecific reduction of cell proliferation was observed at 50 μM.

To identify the lowest effective concentration, transfected COS-7 cells were exposed to CB1954 in the range of 0 to 150 μM (Figure 4B). Cells expressing *gntro *already responded to 1 μM CB1954 with only 41% of living cells 48 hours after prodrug treatment. In contrast, no effect could be detected in *gntr*-expressing cells and significant signs of apoptosis became first evident at 10 μM and 25 μM CB1954, where 39% and 67% of the cells were killed, respectively. However, at these concentrations only about 5% of the *gntro*-expressing cells survived. Comparable ablation for *gntr*-expressing cells were obtained only at 100 μM and 150 μM CB1954, but the number of control cells was also decreased at these concentrations, most likely due to inhibition of cell proliferation as revealed by microscopic analysis (data not shown).

### Codon optimization overcomes tissue-specific codon usage limitations

**Figure 5:**
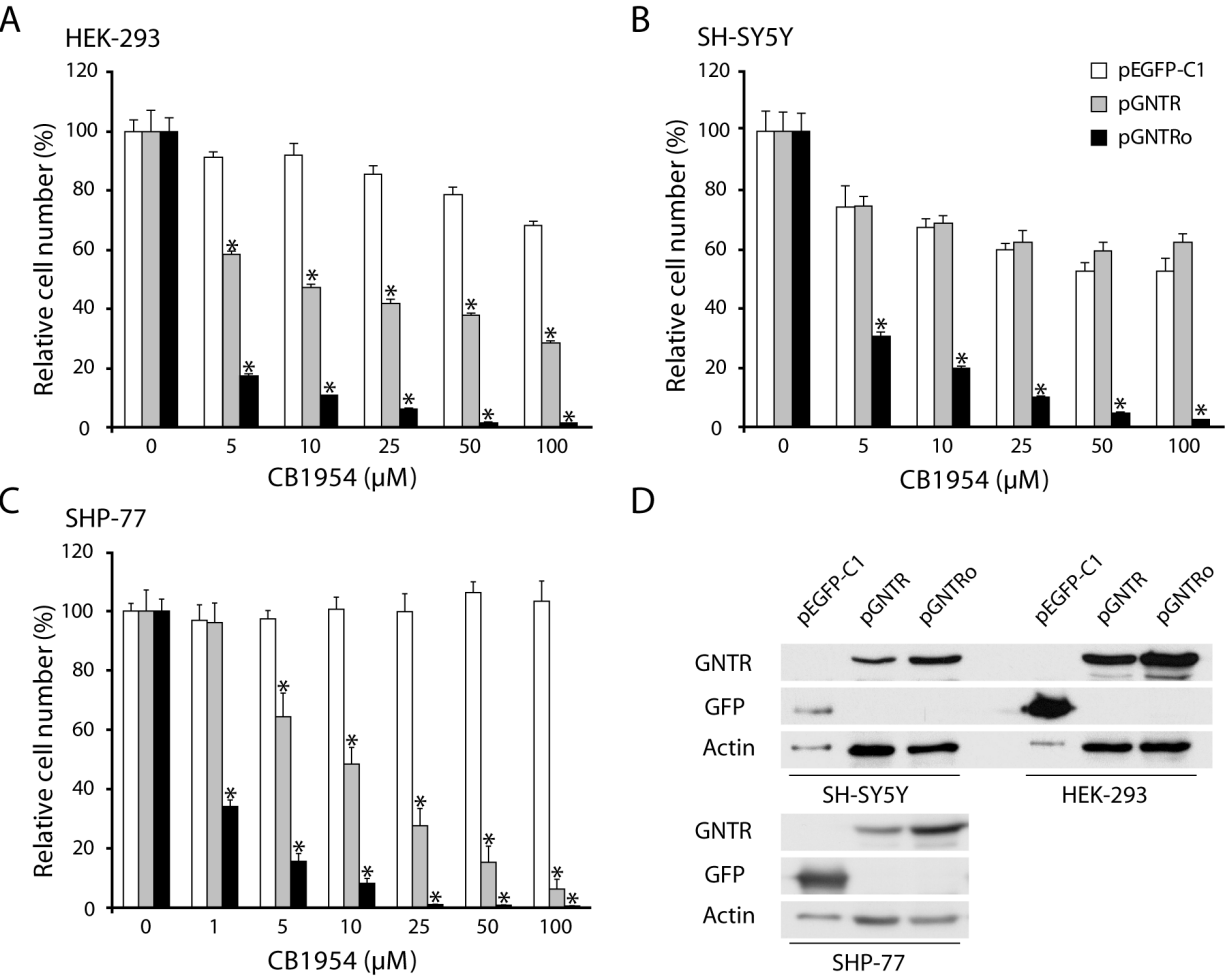
**Codon optimization increases the sensitivity of stable human cell lines to the prodrug CB1954**. (A) HEK-293, (B) SH-SY5Y and (C) SHP-77 cells stably transfected with pGNTR, pGNTRo or pEGFP-C1 (control). MTT assays were performed to determine relative cell viability after 48 hours of cultivation in presence of different CB1954 concentrations. One representative experiment out of three is shown. *: p < 0.05; n = 4. (D) The expression of GNTR was confirmed by immunodetection using anti-GFP and anti-actin (loading control) antibodies. Amount of lysate loaded to each lane: pEGFP-C1 (3 μg), pGNTR (20 μg) and pGNTRo (20 μg).

COS-7 cells represent an artificial system for ectopic gene expression [23]. Thus, stable cell lines from diverse tissues were required to confirm the improvement of the NTR/CB1954 system by codon usage optimization under more native conditions. Therefore, we generated human embryonic kidney (HEK-293), human neuroblastoma (SH-SY5Y), human small cell lung carcinoma (SHP-77), human osteosarcoma (U-2 OS), mouse fibroblast (3T3-L1), and mastocytoma (P815) cell lines stably expressing *gntr*, *gntro *and *gfp *(control) and treated them with different concentrations of CB1954 (Figure 5 and Additional file 2).

Remarkable differences in survival rates were detected in a concentration dependent manner after 48 hours of treatment (Figure 5A, B and 5C). All *gntro*-expressing cell lines showed a dramatic decrease in cell number already at 5 μM CB1954 with only 17%, 30% and 16% of cells still alive as compared with control cells, respectively. Almost no cells survived at 50 μM CB1954. These results were in line with the data obtained from COS-7 cells transiently transfected with *gntro *(Figure 4B). CB1954 also decreased the cell number of *gntr*-expressing HEK-293 and SHP-77 cells dose-dependently, but the sensitivity was clearly lower at all concentrations compared with *gntro*-expressing cells. Indeed, 58% and 64% of *gntr*-expressing HEK-293 and SHP-77 cells were still alive at 5 μM and higher ablation rate of 72% and 94% were only reached at 100 μM, respectively (Figure 5A and 5C). These results were consistent with other stable human (U-2 OS) and mouse (P815, 3T3-L1) cell lines tested (Additional file 2).

Surprisingly, *gntr *expression in SH-SY5Y cells had no effect on their sensitivity to CB1954 (Figure 5B). The observed decrease in cell number of approximately 40% in these cells was not different from *gfp*-expressing control cells and was most likely caused by a CB1954-mediated inhibition of cell proliferation, as revealed by microscopic analysis. These results were consistent with another neuronal cell line (NG-108) tested (data not shown), most likely due to differential tissue-specific codon usage [24,25].

The presence of full-length GNTR protein was analyzed in all cell lines through immunoblotting of whole cellular lysates using an anti-GFP antibody (Figure 5D). The expression of *gntr *was less efficient to that of *gntro *in all three cell lines and correlates with the decreased sensitivity of these cells to CB1954 (Figure 5A, 5B and 5C).

### Codon optimization improves bystander effect

**Figure 6:**
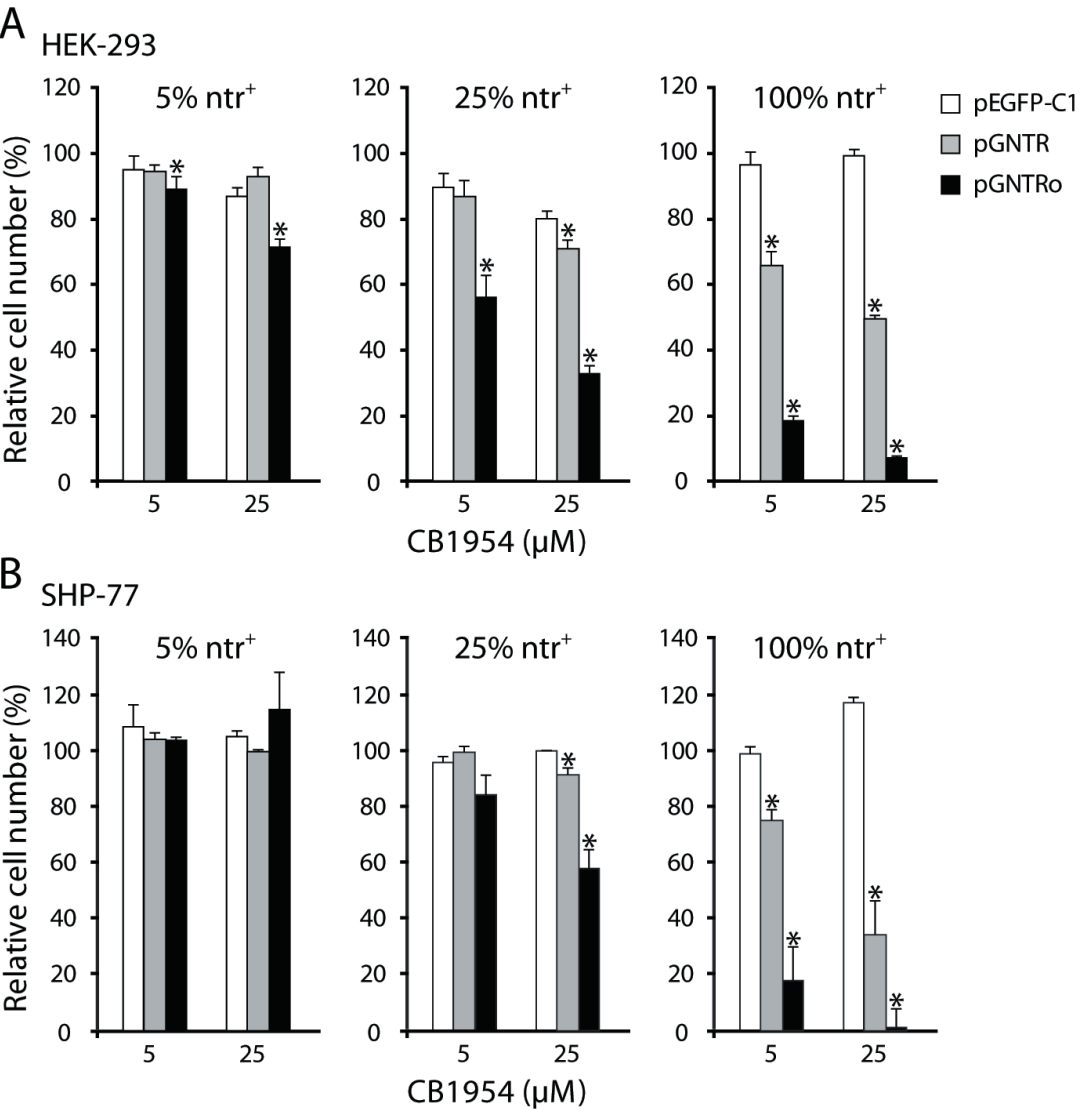
**Codon optimization improves the bystander effect of the NTR/CB1954 system**. (A) At 5% NTR-positive (ntr^+^) HEK-293 cells, only pGNTRo led to a detectable bystander effect, starting at a CB1954 concentration of 5 μM 48 hours after treatment. The effect of pGNTR became detectable at 25% ntr^+ ^cells and 25 μM prodrug. (B) SHP-77 expressing *gntro *showed a significant bystander effect only at 25% ntr^+ ^cells and 25 μM, while *gntr*-expressing cells did not. (A, B) The 100% ntr^+ ^control experiments consistently reproduced the results obtained in Figure 5A and C. The data are normalized to the corresponding CB1954-untreated cell pools. Shown is one representative experiment out of two repetitions. *: p < 0.05; n = 3.

The bystander effect is an important aspect of tumor ablation using GDEPT, which is often desired to overcome limitations of gene delivery to allow effective tumor regression with a low number of suicide gene-expressing cells [26-28]. In order to assess for this effect, various ratios of stably transfected *ntr*-positive (ntr^+^) and wild type HEK-293 cells were mixed and kept at the indicated concentrations of CB1954 (Figure 6). Cell survival and viability was then quantified using a MTT assay after 48 hours of treatment. At 5% ntr^+ ^cells, only *gntro*-expressing cells showed a detectable bystander effect, even at a CB1954 concentration of 5 μM (Figure 6A). A similar effect was detected for *gntr*-expressing cells only at 25% ntr^+ ^cells and 25 μM prodrug (Figure 6A). SHP-77 cells, although responding only at 25% ntr^+ ^and 25 μM (*gntro*), also showed a bystander effect, while *gntr*-expressing cells did not (Figure 6B).

## Discussion

Chemotherapy as one pillar of conventional cancer treatment is associated with severe side effects. GDEPT with prodrug-activating enzymes is a very promising approach for anti-cancer treatment, which provides the opportunity to generate high local cytotoxin concentrations in tumor cells without causing systemic cytotoxicity. Among the systems described so far, alkylating agent-producing systems are believed to be the best candidates for GDEPT approaches in humans because of their independence from cell proliferation [4]. This is particularly important because the number of proliferating cells in human solid tumors is estimated being only about 6% [8]. Especially the NTR/CB1954 system is considered promising, since CB1954-mediated apoptosis is independent from the tumor suppressor gene *p53*, which is inactive in more than 50% of all human cancers [12,29]. For this reason we decided to analyze the expression of bacterial NTR in mammalian cells in order to assess for optimal translational efficacy.

The therapeutical benefits of such enzyme/prodrug systems depend on the cytotoxin-producing activity, which can be obtained in tumor cells. Many efforts have been undertaken to improve the catalytic activity of NTR, for instance by site-directed mutagenesis of the catalytic core [16,17]. A F124K mutant was described, which is 5-fold more potent in sensitizing SKOV3 ovarian carcinoma cells to CB1954 [16]. More recently, a number of new "turbo-NTR" variants were identified by library screening [17]. The most effective T41Q/N71S/F124T-NTR triple mutant showed sensitization of SKOV3 carcinoma cells already at 40- to 80-fold lower CB1954 concentrations than required for the wild type NTR [17]. However, the sensitivity to the prodrug remains limited by the amount of functional NTR protein in the cell.

Codon usage has been shown to impair expression of bacterial genes in eukaryotes [20,21], most probably due to depletion of tRNAs for rare codons. This can delay translational processing and affect cotranslational protein folding of the nascent protein chain. Accordingly, the expression of the bacterial *gntr *gene in COS-7 cells resulted in the formation of cytoplasmic protein aggregates (Figure 2B). Adaptation of the *E. coli*-specific codon usage for GNTR expression in mammals abolished protein aggregation (Figure 2B) and enabled generation of high protein levels in COS-7 cells (Figure 3A), as shown for other bacterial genes [20,21]. As GFP can form dimers at high concentrations, and NTR is also dimeric, the observed aggregation could be also mediated by the GFP portion of the GNTR fusion protein. However, our results rather point to a translational problem, because the much higher *gntro *expression did not lead to such aggregates (Figure 2B).

High dosages of CB1954 caused mild side effects in transgenic mice, for example temporary weight loss of ~10% [13,15,30], most likely due to effects on the intestinal flora (presence of *E. coli *bacteria in the intestinal tract), but also more severe effects, like testis degeneration (unpublished observations). Chung-Faye *et al*. reported no significant toxicity of CB1954 up to 24 mg/m^2 ^in humans, whereas dose-limiting toxicities were seen at 37.5 mg/m^2^, causing diarrhea and hepatic toxicity in phase I clinical trials [31]. In respect of these side effects, the more efficient expression of the synthetic *ntro *gene used in this study allows application of lower CB1954 concentrations *in vitro *(Figure 4 and 5). However, the high efficacy of *ntro *has to be determined *in vivo *in follow-up studies, where lower prodrug concentrations should limit the negative side effects observed in mice.

Our results clearly show the improvement of the NTR/CB1954 enzyme/prodrug system by using a mammalianized version of *ntr*. Most mammals, including humans and mouse, have a very similar codon usage [22]. Thus, our optimized *ntro *gene should also be suitable for human applications. This was demonstrated by the expression of *gntro *in several human cell lines (HEK-293, SHP-77, U-2 OS), which results in higher expression and increased sensitization of these cells to CB1954 compared with *gntr*-expressing cells (Figure 5 and Additional file 2).

Moreover, the codon adaptation leads to an improved bystander effect (Figure 6). Several *in vitro *and *in vivo *studies have proven a potent bystander effect for the NTR/CB1954 system [26-28], which we also detected in *gntr*-expressing COS-7 and HEK-293 cells (Figures 4A and 6A). This is an important aspect of tumor ablation using GDEPT, which is often desired to overcome limitations of gene delivery and low numbers of suicide gene-expressing cells, suggesting higher tumor regression efficacy of the optimized NTR/CB1954 system.

In spite of the previous reports successfully using the NTR/CB1954 system in various applications in the brain [13,14,30], we noticed expression problems of *gntr *in neuronal cell lines resulting in an inefficient response to the prodrug CB1954 (Figure 5B). SH-SY5Y cells stably expressing *gntr *were growth-inhibited but not ablated by CB1954 in contrast to cells expressing the optimized *gntro *gene. Interestingly, HEK-293, SHP-77, U-2 OS, 3T3-L1 and P815 cells either expressing *gntr *or *gntro *were efficiently ablated by the prodrug (Figure 5A and 5C). Plotkin *et al*. (2004) reported differences in synonymous codon usage between genes selectively expressed in six adult human tissues, suggesting that codon-mediated translational control may play an important role in the differentiation and regulation of tissue-specific genes in humans [24]. Furthermore, Dittmar *et al*. reported that isoaccepting tRNAs are differentially expressed in a study spanning eight human tissues [25]. Hence, these findings could explain the differences between non-neuronal and neuronal cells used in our study.

For an efficient application in human cancer treatment GDEPT requires the specific delivery of the prodrug-activating genes to tumor cells, a problem, which has not been solved yet. Many efforts have been undertaken to develop adenoviruses and retroviruses as gene delivery vehicles. Prolonged survival times or even complete cure were observed in murine xenograft models of human cancers [27,32,33]. Furthermore, adenoviral gene delivery of *ntr *was tested in first clinical trials in patients with liver tumors showing the feasibility and tolerance of that approach [34].

## Conclusion

Our data provide evidence that the NTR/CB1954 system can be efficiently improved at the translational level by codon usage optimization, thereby increasing its potential as GDEPT for human cancer. Moreover, codon usage optimization should be also of relevance for other enzyme/prodrug systems. Finally, the codon-optimized *ntro *developed in this study should be also of value for the investigation of cell functions by conditional targeted cell ablation in transgenic animals.

## Competing interests

The authors declare that they have no competing interests.

## Authors' contributions

MG participated in the design of the study, performed most of the experimental work and drafted the manuscript. NP performed FACS sorting, contributed to experimental work, data interpretation and writing the manuscript. SF characterized SHP-77, P815, 3T3-L1 and U-2 OS cell lines. JV participated in the generation of stable cell lines and revision of the manuscript. JP took part in data interpretation and critical revision of the manuscript. DJW was involved in the conception and coordination of this project and writing the manuscript. All authors read and approved the final version of the manuscript.

## Pre-publication history

The pre-publication history for this paper can be accessed here:

http://www.biomedcentral.com/1471-2407/9/301/prepub

## Supplementary Material

Additional file 1 **Codon usage of the bacterial *ntr *vs. the synthetic *ntro *gene**. Critical *E. coli ntr *codons were adapted to murine preferences for high expression in mammalian cells. Click here for file

Additional file 2 **MTT cytotoxicity assays with stable 3T3-L1, U-2 OS and P815 cell lines**. Codon optimization increases the sensitivity of all cell lines to the prodrug CB1954. Click here for file
