# Supplementary material for: A mammalianized synthetic nitroreductase gene for high-level expression
Source: BMC Cancer. 2009 Aug 27;9:301. doi: 10.1186/1471-2407-9-301 (PMC3087338; doi:10.1186/1471-2407-9-301)
Supplement: Additional file 1 — Codon usage of the bacterial ntr vs. the synthetic ntro gene. Critical E. coli ntr codons were adapted to murine preferences for high expression in mammalian cells. [file 1471-2407-9-301-S1.pdf]

Grohmann *et al.* Additional File 1

Additional File 1 Codon usage of the bacterial *ntr* vs. the synthetic *ntro* gene.

			frequency (%)#		number		frequency (%)	
aa		codon	*E. coli*	*M. musculus*	*ntr*	*ntro*	*ntr*	*ntro*
A	Ala	GCT	15	30	4	19	15	70
		GCC	28	42	12	8	44	30
		GCA	21	22	5	-	19	-
		GCG	36	6	6	-	22	-
R	Arg	CGT	35	12	5	-	71	-
		CGC	40	18	2	-	29	-
		CGA	5	11	-	-	-	-
		CGG	11	19	-	-	-	-
		AGA	5	17	-	-	-	-
		AGG	4	22	-	7	-	100
N	Asn	AAT	57	40	1	-	13	-
		AAC	43	60	7	8	88	100
D	Asp	GAT	66	30	13	11	76	65
		GAC	34	70	4	6	24	35
C	Cys	TGT	42	42	1	-	100	-
		TGC	58	58	-	1	-	100
Q	Gln	CAA	35	13	2	-	29	-
		CAG	65	87	5	7	71	100
E	Glu	GAA	62	36	9	5	69	38
		GAG	38	64	4	8	31	62
G	Gly	GGT	30	22	8	-	73	-
		GGC	41	35	3	11	27	100
		GGA	10	21	-	-	-	-
		GGG	18	23	-	-	-	-
H	His	CAT	56	37	4	-	57	-
		CAC	44	63	3	7	43	100
I	Ile	ATT	48	32	2	-	29	
		ATC	45	55	5	7	71	100
		ATA	7	13	-	-	-	-
L	Leu	TTA	14	6	2	-	11	-
		TTG	15	13	-	-	-	-
		CTT	11	12	2	-	11	-
		CTC	12	18	3	3	16	16
		CTA	3	8	1	-	5	
		CTG	45	42	11	16	58	84
K	Lys	AAA	77	35	14	3	78	17
		AAG	23	65	4	15	22	83
M	Met	ATG	100	100	5	5	100	100
F	Phe	TTT	61	34	6	-	55	-
		TTC	39	66	5	11	45	100
P	Pro	CCT	14	26	-	6	-	75
		CCC	6	35	1	2	13	25
		CCA	18	28	1	-	13	-
		CCG	61	11	6	-	75	-
S	Ser	TCT	15	16	2	-	15	-
		TCC	14	26	4	3	31	23
		TCA	11	13	-	-	-	-
		TCG	20	5	1	-	8	-
		AGT	16	13	1	-	8	-
		AGC	25	28	5	10	38	77
T	Thr	ACT	14	24	1	-	9	-
		ACC	47	35	6	11	55	100
		ACA	11	32	-	-	-	-
		ACG	27	9	4	-	36	-
W	Trp	TGG	100	100	3	3	100	100
Y	Tyr	TAT	69	40	1	-	25	-
		TAC	31	60	3	4	75	100
V	Val	GTT	25	14	8	-	40	-
		GTC	18	28	4	-	20	-
		GTA	14	7	2	-	10	-
		GTG	43	51	6	20	30	100
*	Stop	TAA	64	24	1	-	100	-
		TAG	9	41	-	-	-	-
		TGA	27	34	-	1	-	100

adapted from www.kazusa.or.jp
