# Supplementary material for: A mammalianized synthetic nitroreductase gene for high-level expression
Source: BMC Cancer. 2009 Aug 27;9:301. doi: 10.1186/1471-2407-9-301 (PMC3087338; doi:10.1186/1471-2407-9-301)
Supplement: Additional file 2 — MTT cytotoxicity assays with stable 3T3-L1, U-2 OS and P815 cell lines. Codon optimization increases the sensitivity of all cell lines to the prodrug CB1954. [file 1471-2407-9-301-S2.pdf]

# Grohmann *et al.* Additional file 2

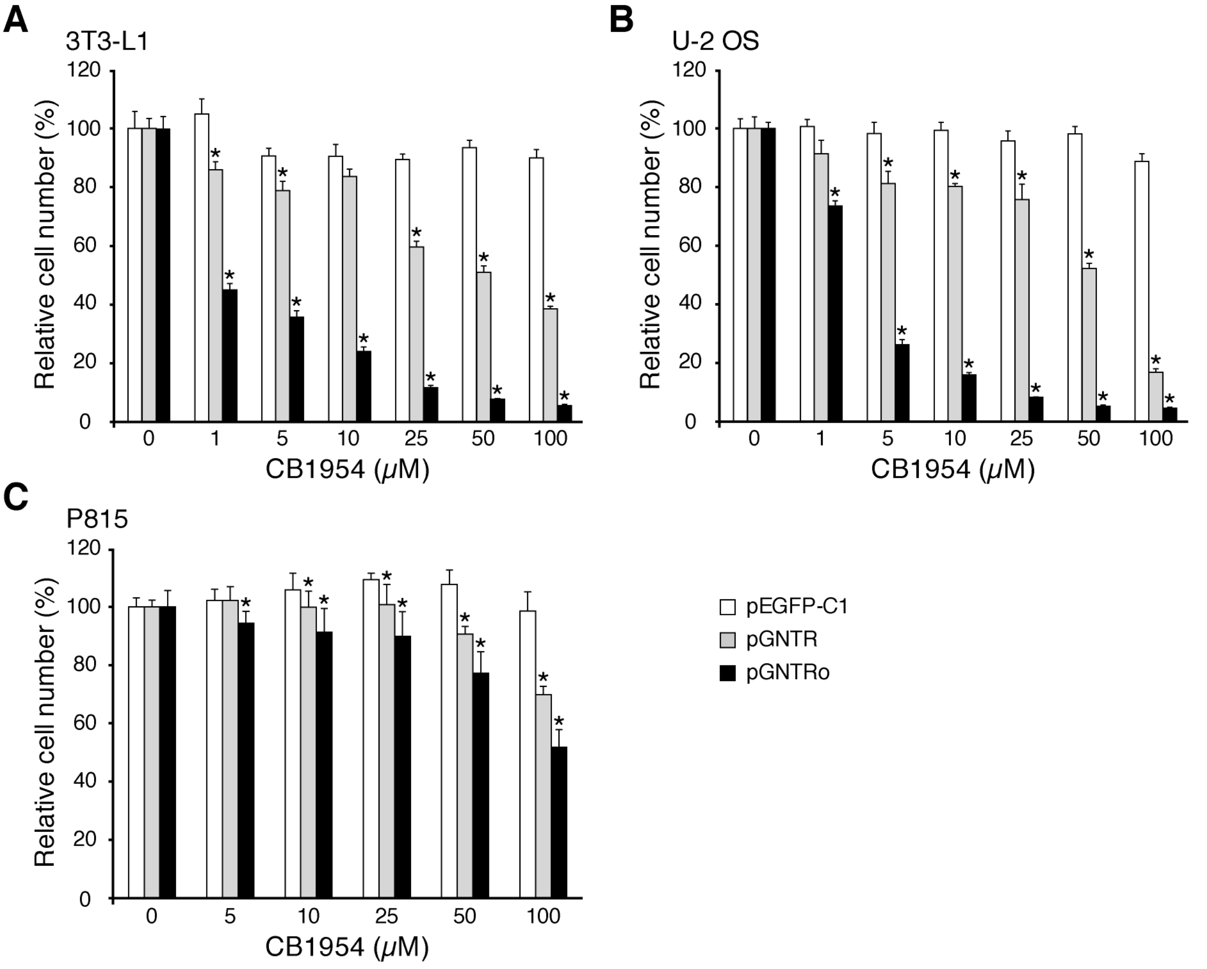


**Additional File 2 - Codon optimization increases the sensitivity of stable human cell lines to the prodrug CB1954.** (A) 3T3-L1, (B) U-2 OS and (C) P815 cells stably transfected with pGNTR, pGNTRo or pEGFP-C1 (control). MTT assays were performed to determine relative cell viability after 48 hours of cultivation in presence of different CB1954 concentrations. (A, B, C) One representative experiment out of two is shown. *: $p < 0.05$; $n = 4$.
